# An Integrated NMR Approach for Evaluating Linker-Payload Conjugation with Monoclonal Antibodies

**DOI:** 10.1021/acs.bioconjchem.6c00017

**Published:** 2026-02-06

**Authors:** Veronica Ghini, Sofia Siciliano, Leonardo Querci, Lorenzo Angiolini, Giuseppina Ivana Truglio, Elena Cini, Mario Piccioli, Elena Petricci, Paola Turano

**Affiliations:** † Department of Chemistry, 9300 University of Florence, via Della Lastruccia 3, 50019 Sesto Fiorentino, Florence, Italy; ‡ Department of Biochemistry, Chemistry and Pharmacy, via A. Moro 2, 53100 Siena, Italy; § Center of Magnetic Resonance, University of Florence, via Luigi Sacconi 6, 50019 Sesto Fiorentino, Florence, Italy

## Abstract

Antibody-drug conjugates (ADCs) are modern biopharmaceuticals that combine the therapeutic effects of small-molecule drugs with the outstanding selectivity of monoclonal antibodies (mAbs). Since their introduction in the biomedical field, research has focused on elucidating the structure, stability, and mode of action of ADCs. Nevertheless, standard characterization methods for ADCs heavily rely on disruptive techniques like mass spectrometry in a non-physiological environment. Here, we present an NMR approach combining ^1^H–^13^C ALSOFAST-HMQC and T_2_-edited ^1^H CPMG experiments, which together provide information on: i. the fingerprint and higher-ordered structure (HOS) of mAbs and ADCs and ii. the properties of the bound linker-payload fragment. In this study, we chose Trastuzumab as a well-known mAb and a Remdesivir-derived fragment as a linker-payload model system to validate our approach.

## Graphical abstract

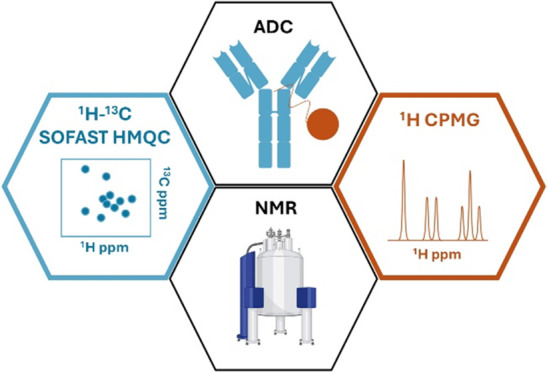

## Introduction

Composed of a monoclonal antibody (mAb) connected via a properly designed linker to a cytotoxic drug called payload, antibody-drug conjugates (ADCs) actually represent one of the most promising classes of molecules for cancer treatment.^1,2^ ADCs combine the specificity of mAbs with the potency of cytotoxic molecules and provide a selective delivery of the payload inside the targeted cell while limiting side effects and improving the overall treatment efficacy. The FDA approval of 15 ADCs^3,4^ is driving the rapid growth of this research field; this also implies an increasing need for effective and robust analytical tools for the characterization of these bioconjugates. The characterization of ADCs primarily relies on the determination and prediction^5,6^ of the drug-to-antibody ratio (DAR) using mass spectrometry (i.e., matrix-assisted laser desorption/ionization, MALDI), possibly coupled with different chromatographic methods.^7^ Nevertheless, there is still a demand for rapid, non-disruptive, and easily accessible methods to establish the efficiency of the bioconjugation process and recognize the difference between aggregates and bioconjugates. While no ADCs have yet been approved for treating viral infections, they represent a promising therapeutic option.

Here, we propose an NMR-based analytical pipeline developed using an ADC based on the commercially available Trastuzumab and the antiviral drug Remdesivir as a model system. This ADC is referred to as B242, with a DAR of about 2.3, i.e., in the optimal range according to results in anticancer ADCs.^8–11^ The approach relies on the characterization of the two components of the ADC, the mAb and the linker-payload system (Figure 1A). Trastuzumab was chosen as an emblematic representative of the mAbs class. Remdesivir is a ProTide prodrug that is converted by esterases, such as Cathepsins A and B, into the active monophosphate nucleoside inside cells (Figure 1B).^12^ The assessment of the higher-order structure (HOS) by NMR is a powerful method to characterize the structural features of mAbs.^13–18^ The methyl-edited ^1^H–^13^C ALSOFAST-HMQC NMR experiment is accepted as the election tool to monitor mAbs stability to stress tests, because the fast rotation of methyl groups around their symmetry axis ensures NMR signals that are well-resolved and intense even for large proteins in natural isotopic abundance.^19,20^ Here, this experiment is used to monitor whether the conjugation reaction to construct the ADC alters the overall structure of the mAb. Then, to selectively observe drug signals in the ADC, we recorded one-dimensional ^1^H CPMG experiments optimized to filter out the broad resonances of the protein (characterized by short T_2_ relaxation, typically in the 10^–4^–10^–3^ s time scale).^21,22^ The signals that arise from the linker-payload system are indeed expected to have long T_2_ relaxation times (10^–1^–1 s). The approach demonstrates its effectiveness in “real-life cases”, i.e., for the characterization of reaction products in systems that are not yet fully purified. Therefore, we propose this integrated experimental approach as a general method applicable to any ADC, beyond the specific case study considered here.

**Figure 1:**
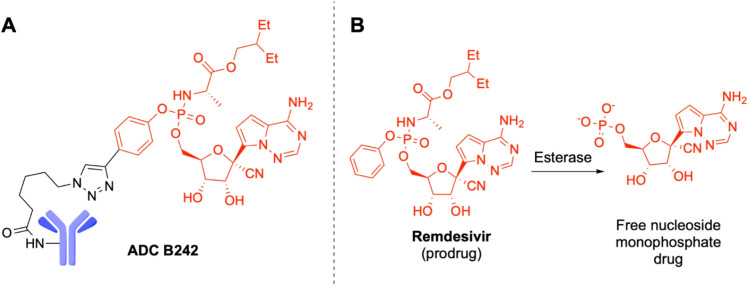
ADC B242. (A) Schematic representation of the mAb (blue) and linker-payload system (black and red, respectively). (B) Activation reaction of Remdesivir by esterase.

## Results and Discussion

### Protein Detected Spectra

#### NMR Fingerprint of Trastuzumab

Trastuzumab is composed of two symmetric units, each of which is made up of 449 amino acids from the heavy chain and 214 amino acids from the light chain, for a total of 663 amino acids. Nuclei in the different units that are related by a symmetry operation are chemically equivalent and will have the same chemical shift, producing a single signal in the NMR spectrum. The resulting spectrum appears therefore to be simplified, as if only one unit were present.

According to the protein sequence of Trastuzumab (Figure 2A), 342 methyl signals are expected corresponding to 220 different amino acids (37 Ala, 47 Leu, 15 Ile, 60 Val, 6 Met, and 55 Thr), well distributed along the protein structure; only 159 could be detected as well-resolved signals in the methyl-edited ^1^H–^13^C ALSOFAST-HMQC (Figure 2B).

**Figure 2:**
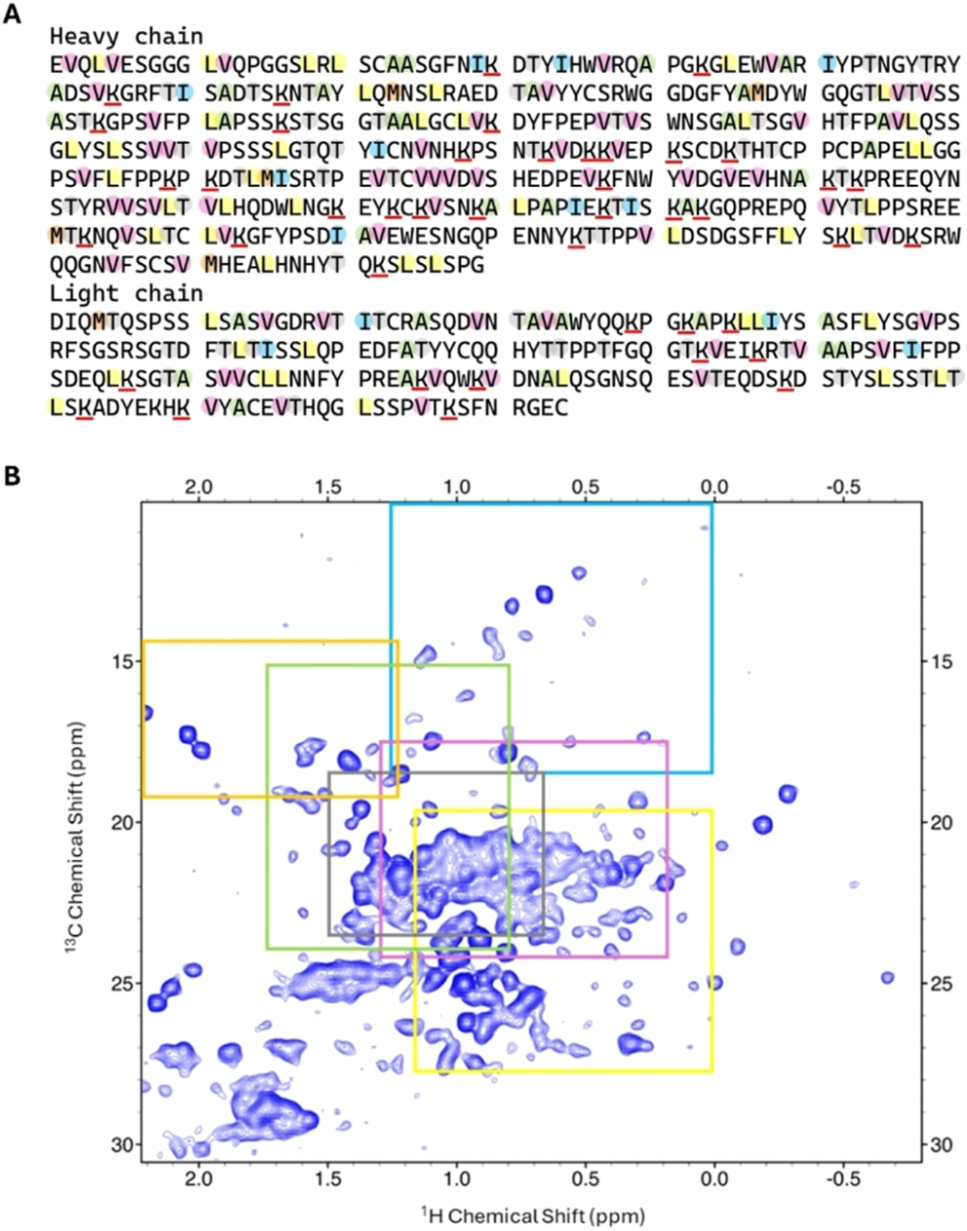
Fingerprint of Trastuzumab. (A) Amino acid sequence of heavy and light chains; the amino acids containing methyl groups are highlighted using the following color code: Val (pink), Leu (yellow), Ala (green), Ile (cyan), and Met (orange); lysine residues are underlined in red. (B) Methyl-edited ^1^H–^13^C ALSOFAST-HMQC spectrum, acquired using a 950 MHz spectrometer at 310 K. Colored squares show chemical shift intervals occurring in BMRB for the specific residues in (A) with frequencies greater than or equal to 5% of the modal class value.

A partial sequence-specific assignment of the single-chain Fab fragment of Trastuzumab has been reported by others (BMRB Entry 52228), which includes the methyl groups of the side chain of 76 amino acids of the Ile, Leu, and Val type.^23^ Based on this assignment, we univocally identified at least one methyl signal for 51 amino acids, as listed in Table S1 (columns “match”); the remaining 25 of these amino acids could not be safely assigned because of spectral crowding in the central region of the spectrum.

To further extend the assignment beyond that available for the Fab fragment, we made reference to the available chemical shift data; Table S2 reports ^13^C and ^1^H chemical shifts for the methyl groups specific for each amino acid type; mean values ± standard deviation were taken from BMRB (https://bmrb.io/). However, it should be noted that their values do not follow a normal distribution but rather a skewed pattern; to avoid biases due to low representative chemical shifts, we used as reference chemical shift ranges those that in the chemical shift distribution histograms, available in BMRB, occur with a frequency greater than or equal to 5% of the modal class value. These ranges are shown in Figure 2B as colored squares.

#### NMR Fingerprint of ADC B242 and Characterization of Binding Sites

The conjugation reaction does not induce striking changes in the protein spectrum, as demonstrated by the comparison of the overall spectral fingerprint obtained by 2D binning of the two ^1^H–^13^C ALSOFAST-HMQC maps recorded for Trastuzumab alone and B242. Indeed, Figure 3A points out a strong correlation (r value of 0.98) between bin intensities in the spectrum of free Trastuzumab and those of ADC B242. It is worth noting that binned two-dimensional maps can be used independently of signal sequence-specific assignment or residue-type attribution and can provide information even in the most crowded areas with severe resonance overlap.

**Figure 3:**
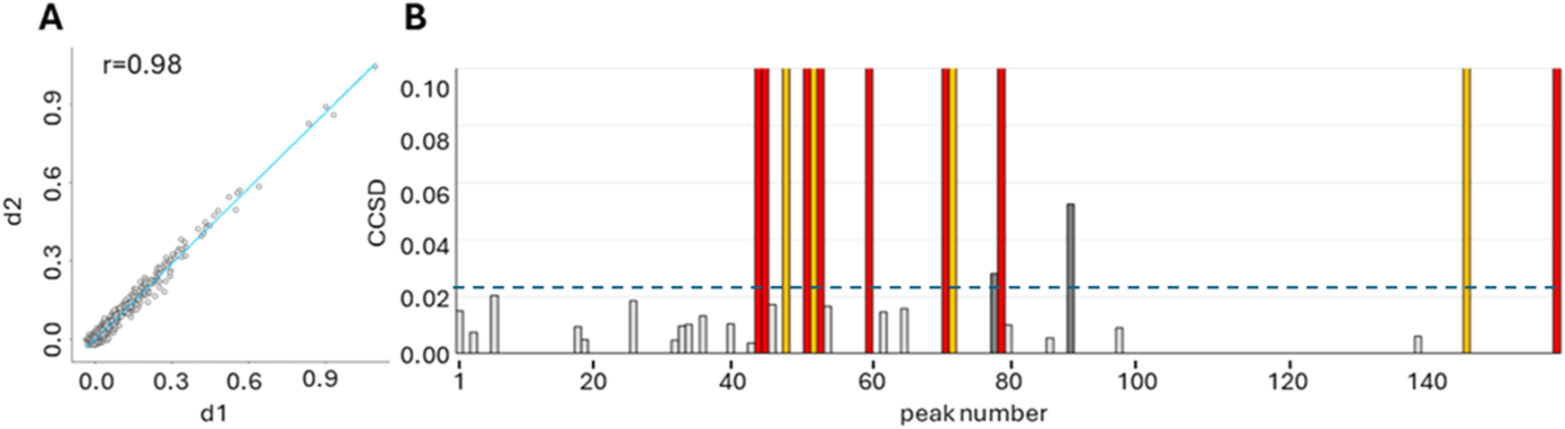
Comparison between free Trastuzumab and ADC B242. (A) Correlation analysis using binned NMR spectra. (B) Combined chemical shift difference (CCSD) analysis, reported as a bar plot with CCSD’s threshold (dashed line) taken as mean + standard deviation (0.024 ppm); CCSD values above the threshold indicate significant changes (dark gray bars), whereas values below the threshold are identified by light gray bars. The red bars represent the methyl peaks that disappeared in the spectrum of ADC B242; the yellow bars represent the methyl peaks that significantly decrease in intensity without undergoing chemical shift perturbations.

Beyond this and even with such a high r value, a few significant localized effects could be detected with signals that (almost) disappear from their original well-resolved position, due to either a decrease in intensity or severe line broadening; no large chemical shift perturbations were observed (Figure 3B).

The most affected signals are labeled in Figure 4A; 8 of them (belonging to 7 distinct amino acid residues) were assigned based on the match with BMRB Entry 52228 (Figure 4B, 2^nd^ column), whereas the other 6 were tentatively attributed to specific residue types based on BMRB chemical shift statistics, as explained above (Figure 4B, 3^rd^ column and Figure S1).

**Figure 4:**
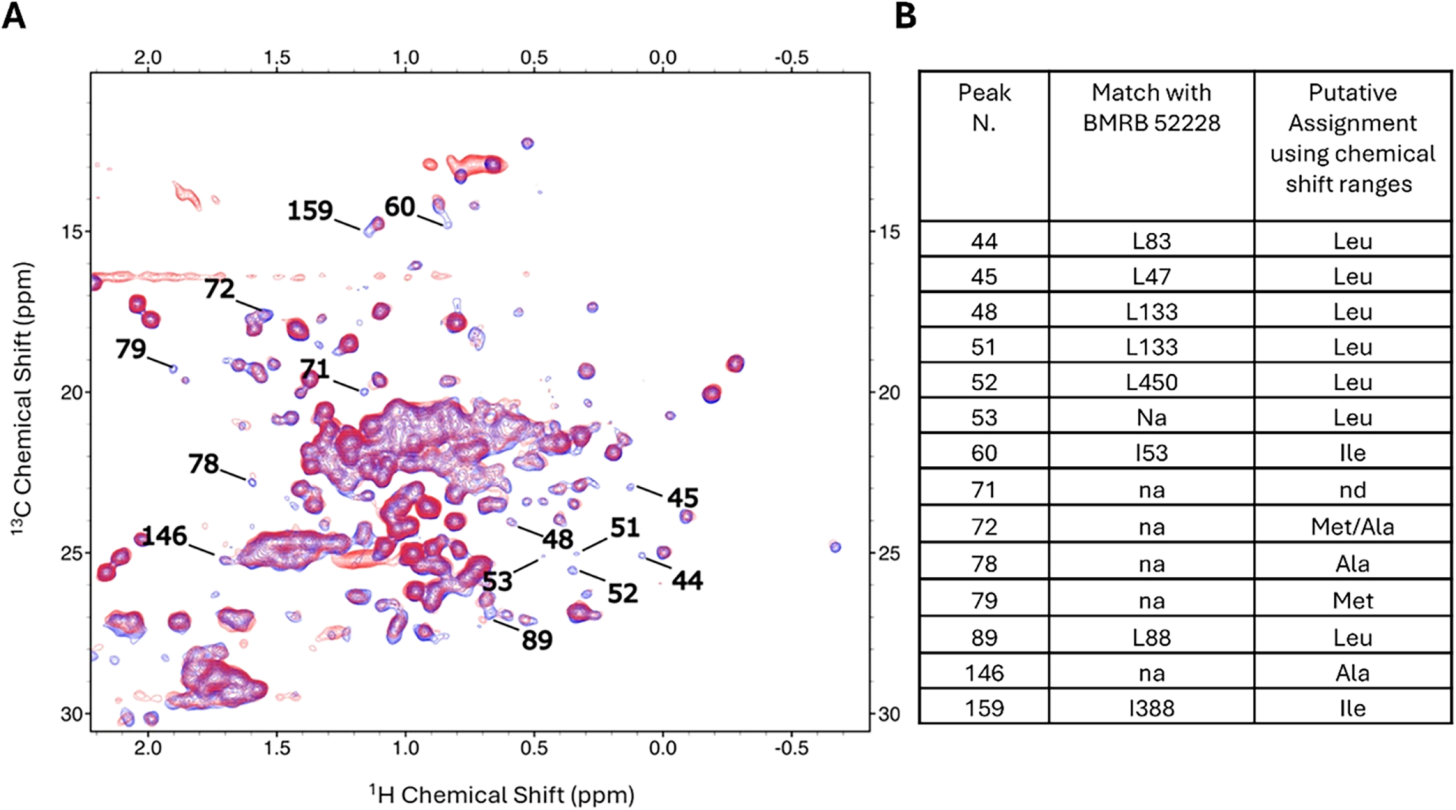
(A) Methyl-edited ^1^H–^13^C ALSOFAST-HMQC spectrum of Trastuzumab (blue spectrum) and B242 (red spectrum) acquired using a 950 MHz spectrometer at 310 K. The most affected peaks are labeled with the respective peak numbers. (B) Table listing the assignment of the most affected peaks using (i) the match with BMRB 52228 (2nd column) and (ii) the chemical shift intervals occurring in BMRB for the specific residues with frequencies greater than or equal to 5% of the modal class value.

Each unit of Trastuzumab contains 44 lysines (Figure 2A), distributed on both the heavy and light chains. Each of them represents a potential reaction point for our linker-payload system. The observed DAR of 2.3 (calculated on the entire antibody) indicates that only a small fraction of them reacts (Supporting Information). Figure 5 summarizes the distribution of the lysine residues on the Fab fragment (PDB code 5XHG),^24^ while (to our knowledge) the coordinates of the entire Trastuzumab are not available. Based on our sequence-specific assignment (BMRB Entry 52228), residues affected by the conjugation reaction are unambiguously located on both the light and heavy chains of the Fab. Therefore, the reaction is not site-specific, and the observed DAR is the result of an average among multiple derivatives.

**Figure 5:**
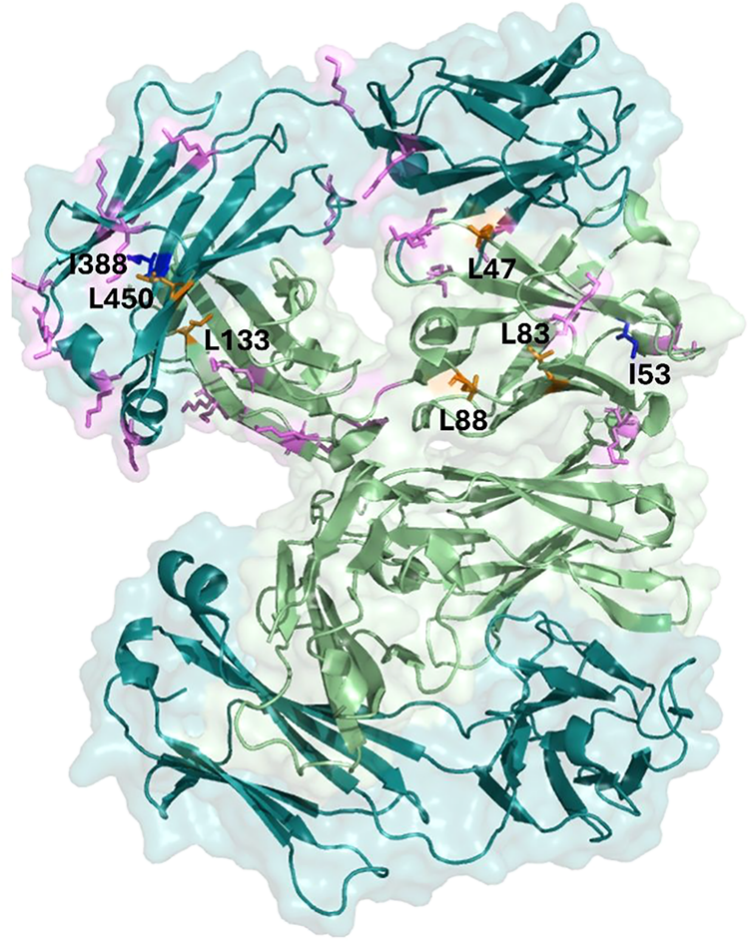
Fab fragment’s structure of Trastuzumab (PDB code 5XHG). The lysine residues are shown as magenta sticks only on one Fab unit. Residues affected by the conjugation reaction with the linker-payload system are shown as blue (isoleucine residues) and orange (leucine residues) sticks.

### Linker-Payload Detected Experiments

The one-dimensional ^1^H NMR spectrum of the free linker-payload system is reported in Figure 6A,B (blue trace) and in Figure S2. The NMR assignment was performed by using ^1^H–^1^H TOCSY experiments (Figures S3–S6). The NMR spectra show evidence of multiple forms, as detailed in the Supporting Information.

**Figure 6:**
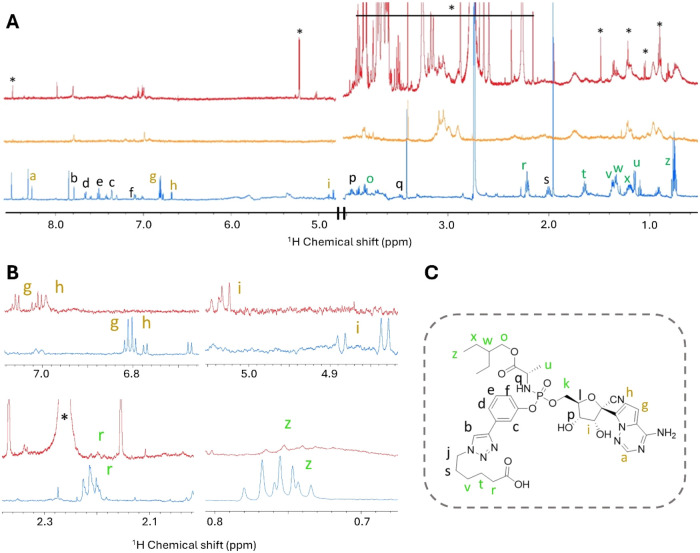
Linker-payload detected experiments. (A) ^1^H CPMG spectra of ADC B242 (red trace), Trastuzumab (orange trace), and free linker-payload system (blue trace). (B) Magnifications of selected regions of the spectra. Signals marked with letters correspond to the hydrogens labeled in (C). (C) Structure of the linker-payload system with hydrogens colored according to the behavior of the respective NMR signals in the ADC B242 spectrum: green, line broadening and negligible chemical shift changes; gold, significant chemical shift changes and negligible line broadening; black, not observable.

Then, we focused on the linker-payload system in the ADC; the one-dimensional ^1^H CPMG NMR spectra are reported in Figure 6A,B (red trace). This experiment was successful in filtering out fast-relaxing protein resonances while enhancing the signals of the small component of the ADC, as shown in Figure S7. The assignment of the free linker-payload system allows us to attribute the non-protein signals observed in the NMR spectra of the ADC; as far as we can assess, the multiple forms already observed for the free linker-payload system are maintained. It should be noted that the bioconjugation product is not highly pure; in fact, signals attributable to impurities were also detected as sharp peaks in the ADC spectrum, as expected for low-molecular-weight contaminants (marked with an asterisk in Figure 6A,B). Nevertheless, no signals attributable to the free linker-payload are observed.

Distinct behaviors are observed for groups of signals of the linker-payload system in the ADC, as summarized in Figure 6:

(i) Signals from the terminal part of the hexanoic acids as well as signals from the alkyl part of the molecule (labeled in green in Figure 6C) are visible in the spectra of the bioconjugate with a negligible chemical shift perturbation, although they are affected by a significant line broadening;
(ii) All signals from the aromatic region bound to the carboxylic acids (labeled in black in Figure 6C) are not visible, indicating the occurrence of interactions between this part of the linker-payload system and the mAb that cause signal line broadening beyond detection;
(iii) Signals attributable to the nucleoside group do not show significant line broadening, but significant chemical shift perturbations (labeled in gold in Figure 6C). This suggests that conformational changes in this portion of the molecule occur when the linker-payload system is bound to the antibody.

## Conclusions

The common approach adopted in ADC chemistry to derive DAR values is the use of MALDI MS,^25^ which offers superior sensitivity with respect to NMR. Nevertheless, this information is not site-specific. Identifying drug bioconjugation sites via MS usually requires digestion.^25,26^ This procedure is challenging for ADCs due to missed cleavages caused by steric hindrance of the linker-payload.^27^ Additionally, the linker-payload can make peptides hydrophobic, causing them to stick together and making them difficult to recover.

On the other hand, NMR has established as a powerful tool to evaluate the HOS of protein therapeutics,^28^ which has no counterpart in MS. Indeed, HOS is generally stabilized by weak interactions (hydrogen bonds, and electrostatic and hydrophobic interactions), which can be easily affected during protein expression, purification, formulation, and storage. Given the high sensitivity of NMR chemical shifts to the local environment and of line widths to global and local dynamics, binned ^1^H–^13^C ALSOFAST-HMQC maps can be used to monitor even subtle changes occurring in the mAb structure, regardless of the availability of signal assignment.

The presently proposed combination of HOS and CPMG provides a one-sample tool to monitor the consequences of the reaction both on the mAb side and linker-payload site, respectively. The HOS information becomes site-specific when enough assignment is available to monitor different parts of the mAb structure without requiring any sample fragmentation. The method is effective even at very low DAR, as exemplified in the Supporting Information for the ADC B238, which is made by Trastuzumab and a Doxorubicin payload connected through a more traditional noncleavable linker (DAR 1.3; Supporting text and Figure S8).

The examples here presented refer to sample concentrations in the 110–50 μM range. By lowering the concentration, the signal-to-noise ratio of NMR experiments decreases, making it more difficult to distinguish signals that are broadened beyond detection as a consequence of the bioconjugation reaction from those that simply fall below the detection limit of the 2D experiment. Additionally, the measurement of chemical shift differences is increasingly affected by errors. In our hands, a lower limit of ∼50 μM is established for the HOS part. As recently reported by other authors, attention should be paid to the fact that sample aggregation can induce nonspecific line broadening in the HOS NMR spectra of ADCs with high DAR and/or in the case of particularly hydrophobic linker-payload systems.^29^

The use of T_2_-edited CPMG experiments on the intact ADC provides a tool to monitor, with a simple 1D spectrum, the stability of the linker and of the attached payload under different solution conditions. ADC integrity is crucial because it ensures the delicate balance between targeted delivery and payload release, directly impacting efficacy and safety.

In summary, we have shown here that the combination of ^1^H–^13^C ALSOFAST-HMQC and T_2_-edited ^1^H CPMG experiments could be a general method applicable to any ADC, independently of the DAR values or the nature of conjugation sites. Linker-payload detected experiments contribute to the characterization of the groups interacting with the antibody, while HOS experiments are reporters of the overall stability of the mAb and, eventually, site-specific reporters of the conjugation sites. In principle, this approach can be used for any biologics based on a carrier protein (and not just mAbs) bound to small payloads via flexible linkers, allowing to characterize the former via HOS approaches and to exploit the intrinsic mobility of the linker-payload via CPMG experiments.

## Experimental Procedures

### Production of the ADC

The synthetic work and standard characterization were carried out as detailed in the Supporting Information. Briefly, the linker-payload system was activated with *N*-hydroxysuccinimide, and the resulting reagent was bioconjugated with Trastuzumab lysine residues. The DAR was determined to be 2.3 by MALDI as detailed in the SI.

### NMR Samples Preparation and Experiments

#### Protein Detected Experiments

Samples of Trastuzumab and ADCs were prepared for NMR by diluting stock solutions to a final volume of 350 μL (in shaped NMR tubes), using 90% PBS and 10% D_2_O. The final concentration for Trastuzumab and ADC B242 was 110 μM. Methyl-edited ^1^H-^13^C-ALSOFAST-HMQC experiments were acquired on a Bruker Avance spectrometer at 950 MHz equipped with a TCI cryoprobe, at 310 K. Spectra were recorded with the following parameters: a matrix of 1024 × 160 data-points was collected with 1024 transients per increment. The acquisition times in direct and indirect dimensions were 33 and 13 ms, respectively. The recycling delay for the experiment was 350 ms.

#### Linker-Payload Detected Experiments

One-dimensional ^1^H NMR spectra were acquired for Trastuzumab, ADC B242, and the free linker-payload system (∼30 μM, 90% PBS, 10% D_2_0, 3 mm NMR tube). One-dimensional NOESY and T_2_-edited CPMG experiments were recorded at a 600 MHz Bruker Avance spectrometer equipped with a TXI probe, at 310 K. Spectra were acquired with the following parameters: 3.67 and 4 s for acquisition and recycle delays, spectral width of 20 ppm, and number of scans of 8 K. The optimized spin-lock relaxation delay to suppress mAbs signals in the T_2_-edited ^1^H CPMG experiment was 50.7 ms; this was achieved using 300 μs as echo-delay in the spin echo sequence and 80 repetitions of the spin–echo block.

#### Assignment of Linker-Payload System

The assignment was performed using ^1^H–^1^H TOCSY experiments (with different TOCSY spin-lock times) in combination with the analysis of peak multiplicities and chemical shift predictions (https://www.nmrdb.org/). ^1^H NMR spectra of free linker-payload system (60 μM, 90% PBS, 10% D_2_0, 3 mm NMR tube) were acquired using a 700 MHz Bruker spectrometer, equipped with a TXO cryoprobe, at 310 K. ^1^H–^1^H TOCSY spectra were recorded with the following parameters: a matrix of 2048 × 280 data-points was collected with 128 transients per increment. Acquisition times in direct and indirect dimensions were 143 and 19.6 ms, respectively. The relaxation delay was 2 s, and the TOCSY spin-lock times were 0.05, 0.08, or 0.1 s.

All of the NMR spectra were processed using Topspin 4.0.8 software.

### Data Analysis

Binned 2D NMR spectra were used to compare the overall fingerprint^17,30^ of ADC B242 with respect to Trastuzumab. Spectral binning was performed by using the Amix software. To this end, each spectrum in the region −1.0 to 2.2 ppm (^1^H dimension) and 8.0 to 26.0 ppm (^13^C dimension) was segmented into bins of 0.02 and 0.5 ppm for the ^1^H and ^13^C dimensions, respectively; the corresponding spectral areas were integrated. Prior to statistical analysis, the binned data were normalized using total area normalization. Correlation analysis was performed using the *cor* function in the R software.

Peak picking was performed manually using CARA software. The combined chemical shift difference, hereafter CCSD, was calculated as follows, according to previous reports:^17^

$$\mathrm{CCSD}=\sqrt{{\frac{1}{2}[\left(\delta_{H}-\delta_{\mathrm{Href}}\right)}^{2}+{\left(0.251\delta_{C}-{0.251\delta }_{\mathrm{Cref}}\right)}^{2}]}$$

where δ_H_ and δ_C_ are the ^1^H and ^13^C chemical shifts, respectively, of the analyzed peaks in ADC B242, and δ_Href_ and δ_Cref_ are the ^1^H and ^13^C chemical shifts, respectively, for the same peaks in free Trastuzumab, taken as reference.

### Safety/Hazard Statement

No unexpected or unusually high safety hazards were encountered.

## Supplementary Material



## Notes

Supporting methods for (i) synthesis and characterization of the ADCs: MALDI analysis of bioconjugates, synthesis and characterization of the linker-payload systems, bioconjugation reaction with equation for DAR calculation; (ii) linker-payload NMR resonance assignment in PBS of ADC B242; (iii) NMR characterization of ADC B238; putative assignment of the most affected signals listed in Figure 4, using the chemical shift intervals occurring in BMRB (Figure S1); 1D spectrum of the linker-payload system for B242 (Figure S2); TOCSY spectral regions of B242 (Figure S3–S6); 1D CPMG spectrum of Trastuzumab with different spin-lock times (Figure S7); characterization of ADC B238 (Figure S8); HPLC analysis of compound 7 (Figure S9); assignment of methyl groups in Trastuzumab (Table S1); and representative ^13^C and ^1^H chemical shift regions for methyl groups of each amino acid, taken from BMRB statistics (Table S2) (PDF)
