# Supplementary material for: An Integrated NMR Approach for Evaluating Linker-Payload Conjugation with Monoclonal Antibodies
Source: Bioconjug Chem. 2026 Feb 6;37(2):472–8. doi: 10.1021/acs.bioconjchem.6c00017 (PMC12921666; doi:10.1021/acs.bioconjchem.6c00017)
Supplement: Supplementary file 1 [file bc6c00017_si_001.pdf]

An integrated NMR approach for evaluating linker-payload conjugation with monoclonal antibodies

Veronica Ghini[1], Sofia Siciliano[2], Leonardo Querci[1], Lorenzo Angiolini[2], Giuseppina Ivana Truglio[2], Elena Cini[2], Mario Piccioli[1,3], Elena Petricci[2], Paola Turano[1,3]

[1] Department of Chemistry, University of Florence, via della Lastruccia 3, 50019 Sesto Fiorentino, Florence, Italy.
[2] Department of Biochemistry, Chemistry and Pharmacy, via A. Moro 2, 53100 Siena, Italy.
[3] Center of Magnetic Resonance, University of Florence, via Luigi Sacconi 6, 50019 Sesto Fiorentino, Florence, Italy.

Supplementary Information

Supplementary Text

Synthesis and characterization of the ADCs

Experimental part

General methods

All reagents were used as purchased from commercial suppliers without further purification, if not differently specified. Solvents were dried and purified by conventional methods prior use or, if available, purchased in anhydrous form. For the reactions carried out under anhydrous conditions, all glassware and syringes were dried overnight in an oven thermostated at 140 °C and allowed to cool under vacuum. For the transfer of some reagents sterile packaged one-way plastic syringes were employed. Hypodermic needles for medicinal use were used together with metal needles (specially to transfer anhydrous solvents) and the latter dried in an oven as described previously. The inert gas used for all reactions were nitrogen or argon. Vacuum distillations were accomplished with standard equipment using oil pumps. Flash column chromatography was performed with Merck silica gel 60, 0.040-0.063 mm (230-400 mesh). Merck aluminium backed plates pre-coated with silica gel 60 (UV254) were used for analytical thin layer chromatography and were visualized by staining with a $KMnO_4$ solution. Products purification was carried out also using a 55 µm, 70 Å Strata® SCX column from Phenomenex (Torrance, CA, USA) (phase: 2 g/12 mL strong cation exchange, SCX-SO_3H).

MALDI analysis of bioconjugates

Super DHB was used as matrix, at two different concentrations and both were used in parallel with the same sample: 20.0 mg or 25.0 mg of Super DHB were dissolved in a solution of 150 µl of MeCN, 350 µl of H_2O and 0.05 µl of TFA. Usually, the matrix solutions were freshly prepared. The stainless-steel target was placed in a termoblock setting to 39 °C and then 1.35 µl of the sample was deposited using a micropipette. After the evaporation of the solvent and when the sample was dried, 1.65 µl of matrix solution was deposited. The formation of the crystal, at this plate temperature, requires about 3-5 minutes. Once it was completely dried, it was possible to remove the target from the termoblock. To acquire the sample spectra, the target plate was inserted into the MALDI-TOF instrument and appropriate instrumental parameters were chosen. The unconjugated antibody was always used as a blank. In addition, a calibrant standard, BSA 10 nM, was deposited in the target plate. According to the manufacturer's protocol, bioconjugate samples were desalted using PD SpinTrap-G25 (5000 MWCO, CytivaR). For the preparation of the thin layer, a saturated solution of sinapinic acid in

ethanol was deposited onto the target plate and left to dry. The sample solution and the matrix solution (sinapinic acid 20 mg/mL, acetonitrile/water 0.1% TFA (70/30)) were mixed in a 1:1 ratio. Subsequently, 1 μL of the mixed solution was spotted onto the target plate and left to dry. For every bioconjugate, two sample spots were prepared from the same solution. The spots on the target were chosen so that all samples were near the reference antibody. MALDI-TOF measurements were performed on a Brüker UltrafleX TOF/TOF in linear mode, voltage polarity POS, and 10000 laser shots were accumulated to obtain a spectrum. The intact mass of each Lys bioconjugate was calculated on the doubly charged peak after smoothing with a Savitzky-Golay filter with 50 points for the entire peak.

ADC B242: synthesis of the linker-payload system

The synthesis of the ADC B242 is reported in **Scheme 1** and detailed below.

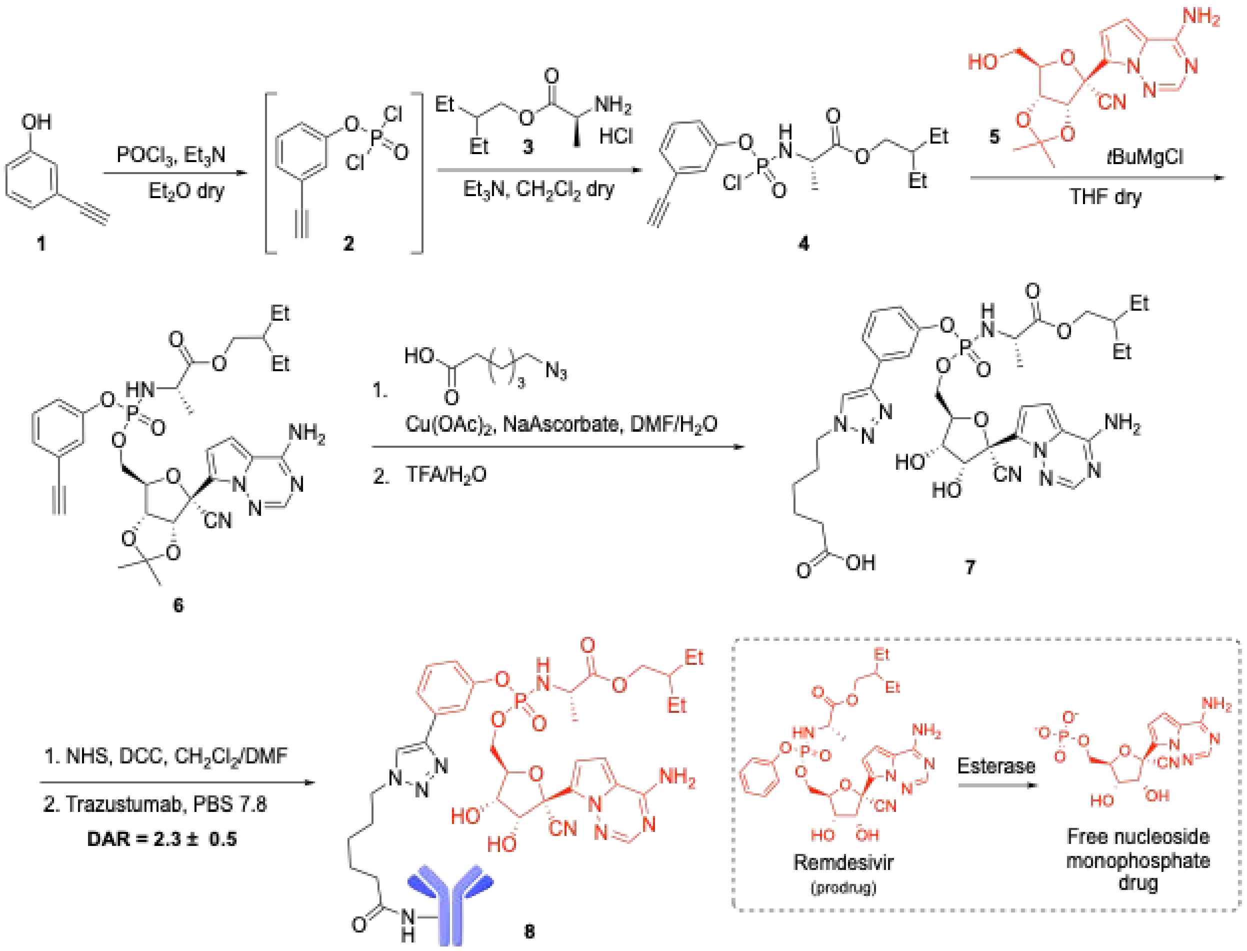

Scheme S1. *Synthesis and bioconjugation reaction of Trastuzumab with Remdesivir-derived linker-payload system.*

6-Azidohexanoic acid

6-Bromo-esanoic acid (300 mg, 1.54 mmol) was solubilized in dry DMF (5 mL) under N_2 and NaN_3 (500 mg, 7.70 mmol) was added. The mixture was stirred at 100 °C for 16h. The crude was diluted with EtOAc (5 mL) and filtered on Büchner and washed with EtOAc (2 x 5 mL). The solution was washed with $KHSO_4$ (2 x 10 mL), H_2O (10 mL) and brine (10 mL). Organic layer was dried over dry Na_2SO_4, filtered and the solvent was removed under vacuum. Compound **9** was obtained as yellow oil with 98% yield (236 mg).

MS (ESI): m/z 156 $[M-H]^-$

^{1}H-NMR (400 MHz, $CDCl_3$) δ 3.19 (t, J = 6.8 Hz, 2H), 2.28 (t, J = 7.4 Hz, 2H), 1.68 – 1.47 (m, 4H), 1.44 – 1.26 (m, 2H).

***2-ethylbutyl ((((3aR,4R,6R,6aR)-6-(4-aminopyrrolo[2,1-f][1,2,4]triazin-7-yl)-6-cyano-2,2-dimethyltetrahydrofuro[3,4-d][1,3]dioxol-4-yl)methoxy)(3-ethynylphenoxy)phosphoryl)-L-alaninate* (6)**

3-ethynylphenol (101 µL, 0.94 mmol) was dissolved in dry Et_2O (2 mL) in a Schlenk tube under Ar atmosphere. The solution was cooled at -78 °C, a solution of dry $POCl_3$ (70 µL, 0.72 mmol) in dry Et_2O (1 mL) and one of dry Et_3N (100 µL, 0.72 mmol) in dry Et_2O (1 mL) were added dropwise. A yellowish precipitate was immediately formed. The mixture was stirred at r. t. for 1 h. The crude was filtered on silica pad under Ar and washed with Et_2O (2 x 10 mL). The solvent was removed under vacuum at 25 °C. Compound **2** (0.72 mmol) was dissolved in dry CH_2Cl_2 (2 mL) and (*S*)-1-(2-ethylbutoxy)-1-oxopropan-2-aminium chloride **3** (150 mg, 0.72 mmol) was added; the solution was cooled at -78 °C and a solution of Et_3N (200 µL, 1.44 mmol) in CH_2Cl_2 (1 mL) was added dropwise. The suspension was stirred at -78 °C for 1 h, than stirred at r.t. and for additional 30 min under Ar. The solvent was removed under *vacuum*, in Ar atmosphere at 25 °C. Et_2O (30 mL) was added to the oil obtained and the mixture was filtered on silica pad under Ar. Et_2O was removed under vacuum at 25 °C and a yellow-green oil was obtained. Reaction was considered quantitative, and the product, kept under Ar, was used in the next step without further purification. Under Ar, to a solution of nucleoside **5** (60 mg, 0.18 mmol) in dry THF (3 mL), *t*BuMgCl 1Min Me-THF (220 µL, 0.22 mmol) was added at 0 °C dropwise and the solution was stirred for 10 min. Phosphoramidate **4** (0.54 mmol) in dry THF (2 mL) was added. The mixture was stirred at r. t. for 3 h. Silica gel (350 mg) was added, and the solvent was removed under vacuum. The crude was immediately purified by flash chromatography on silica gel (gradient 0-10% MeOH in CH_2Cl_2). The expected product was obtained with 86% yield (103 mg).

MS (ESI): 667 $[M+H]^+$; 689 $[M+Na]^+$

^{1}H and ^{13}C refer to the mixture of diastereoisomers.

^{1}H-NMR (400 MHz, CD_3OD) δ 7.90 (s, 1H), 7.27 – 7.18 (m, 3H), 7.11 – 7.06 (m, 1H), 7.02 (d, *J* = 4.7 Hz, 1H), 6.93 (t, *J* = 3.8 Hz, 1H), 5.23 (d, *J* = 6.6 Hz, 1H), 5.00 – 4.93 (m, 1H), 4.63 – 4.59 (m, 1H), 4.38 – 4.27 (m, 2H), 4.05 – 3.92 (m, 2H), 3.87 – 3.78 (m, 1H), 3.53 (s, 1H), 1.68 (s, 3H), 1.50 – 1.21 (m, 14H), 0.85 (t, *J* = 7.5 Hz, 6H).

^{13}C-NMR (151 MHz, CD_3OD) δ 173.62, 165.47, 153.73, 152.40, 150.49, 129.40, 128.34, 123.81, 123.24, 121.26, 120.85, 120.63, 116.41, 115.41, 111.83, 111.62, 92.03, 84.52, 83.63, 81.77, 81.54, 81.22, 78.13, 66.72, 66.51, 65.57, 40.35, 37.40, 24.10, 22.84, 18.94, 9.95.

***6-(4-(3-(((((2R,3S,4R,5R)-5-(4-aminopyrrolo[2,1-f][1,2,4]triazin-7-yl)-5-cyano-3,4-dihydroxytetrahydrofuran-2-yl)methoxy)(((S)-1-(2-ethylbutoxy)-1-oxopropan-2-yl)amino)phosphoryl)oxy)phenyl)-1H-1,2,3-triazol-1-yl)hexanoic acid* (7)**

ProTide **6** (85 mg, 0.13 mmol) and 6-azidohexanoic acid **7** (16 mg, 0.10 mmol) were solubilized in dry DMF (2 mL) under Ar and the solution was subjected to three cycles of Ar/vacuum. To this solution, a previously degassed freshly prepared aqueous mixture (1 mL) of $Cu(OAc)_2$ (6 mg, 0.03 mmol) and sodium ascorbate (12 mg, 0.06 mmol) was added. The reaction mixture was stirred at r.t. for 24 h. The solvent was removed under vacuum, and the crude was purified by flash chromatography (gradient 0-10% MeOH in CH_2Cl_2) obtaining 65 mg (79%) of the expected product. The compound obtained (35 mg, 0.04 mmol) was dissolved in a 0.1M TFA/H_2O 8/2 mixture (400 μL) and stirred for 3 h at r.t.. The solvent was removed under vacuum and the mixture was purified by flash chromatography (gradient 0-10% MeOH in CH_2Cl_2) providing 25 mg (75%) of desired product **7**.

MS (ESI): 784 $[M+H]^+$; 806 $[M+Na]^+$; 782 $[M-H]^-$.

^{1}H and ^{13}C refer to the mixture of diastereoisomers:

^{1}H-NMR (600 MHz, CD_3OD) δ 8.36 – 8.24 (m, 1H), 7.87 – 7.77 (m, 1H), 7.69 – 7.59 (m, 2H), 7.43 – 7.31 (m, 1H), 7.21 – 7.09 (m, 1H), 6.95 – 6.78 (m, 2H), 4.50 – 4.39 (m, 5H), 4.05 – 3.86 (m, 5H), 2.30 (t, *J* = 7.2 Hz, 2H), 2.04 – 1.90 (m, 2H), 1.70 – 1.62 (m, 2H), 1.36 – 1.25 (m, 8H), 0.90 – 0.80 (m, 6H).

^{13}C-NMR (151 MHz, CD_3OD) δ 155.76, 151.21, 147.94, 132.18, 129.89, 124.08, 121.78, 120.15, 119.69, 119.08, 117.17, 116.26, 110.95, 110.74, 101.26, 88.37, 83.05, 82.99, 82.89, 82.83, 79.81, 74.25, 73.50, 70.21, 70.12, 66.71, 66.03, 65.52, 50.22, 50.15, 49.89, 40.31, 29.53, 25.59, 24.02, 22.81, 19.06, 9.91.

HPLC-MS: solvent A (H_2O+ 0.1% HCOOH)/ solvent B (MeCN + 0.1% HCOOH). Gradient 20-80% B/A in 26 min. Mixture of two diastereomers with RT 8.33 (**Figure S8**).

ADC B238: *synthesis of the linker-payload system.*

The synthesis of the ADC B238 is reported in **Scheme 2** and detailed below.

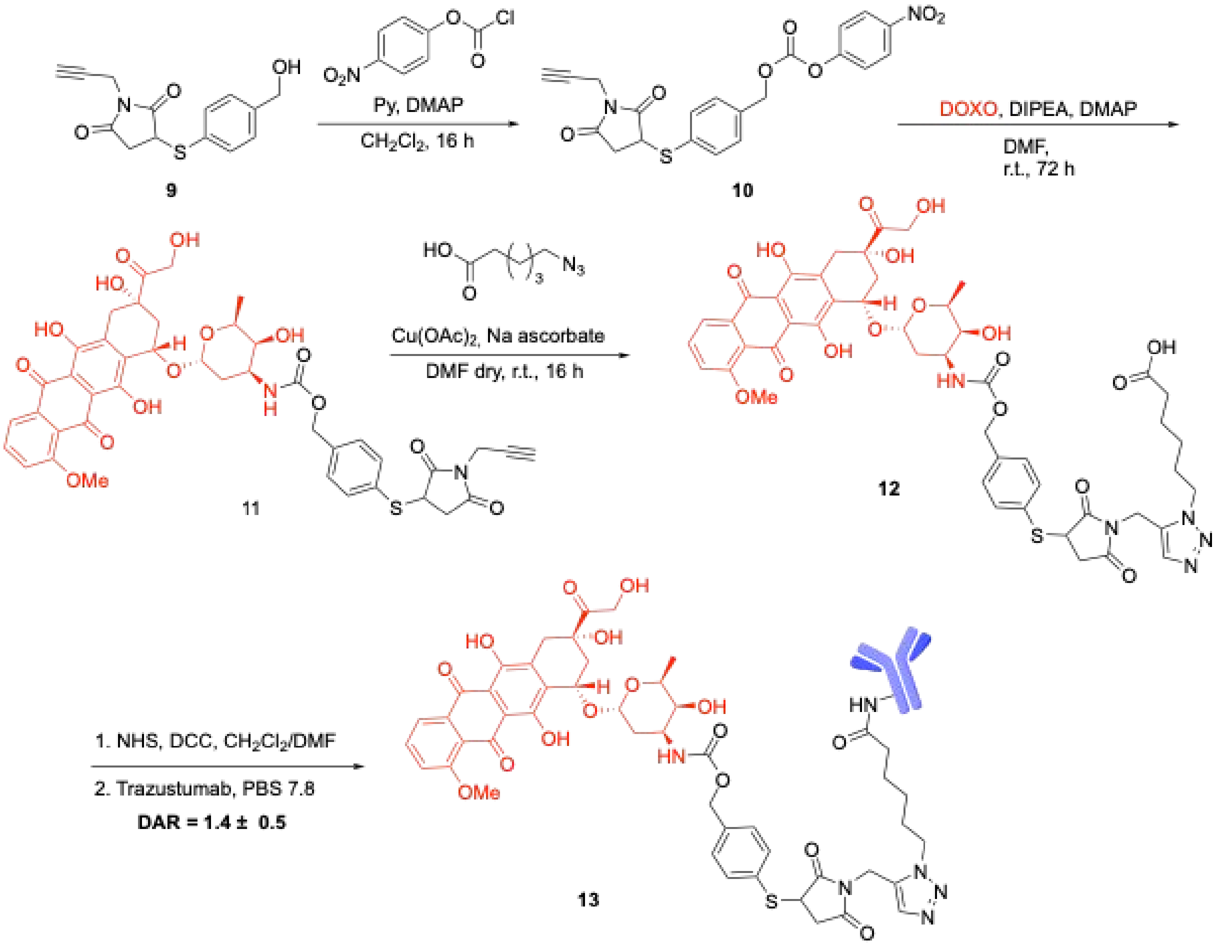

Scheme S2. *Synthesis and bioconjugation reaction of Trastuzumab with Doxorubicin-derived linker-payload system.*

3-((4-(hydroxymethyl)phenyl)thio)-1-(prop-2-yn-1-yl)pyrrolidine-2,5-dione (9)

Maleic anhydride (700 mg, 7.14 mmol) and propargylamine (0.5 mL, 7.85 mmol) were dissolved in glacial acetic acid (14 mL) and stirred at r.t. for 16 h. The reaction mixture was concentrated under reduced pressure, the residue suspended in acetic anhydride (5 mL) and NaOAc (311 mg, 3.57 mmol) was added. The reaction vessel was heated at 65 °C for 2 h. After cooling to r.t., the reaction mixture was diluted in H_2O (70 mL) and extracted with Et_2O (3 × 100 mL). The combined organic phases were dried over Na_2SO_4, filtered, and evaporated under vacuum. The compound was purified by

means of chromatography on silica gel with MPLC Syncore® Büchi eluting 0-50 % gradient of EtAOc in petroleum ether, as a transparent oil (582 g, 4.28 mmol, 60% yield).

^{1}H NMR (400 MHz, $CDCl_3$, δ ppm, *J* Hz) δ 6.66 (s, 2H), 4.12 (s, 2H), 2.13 (s, 1H).

The product obtained (600 mg, 4.36 mmol) and the thiol **72** (350 mg, 2.57 mmol) were solubilized in MeCN (15 mL) and a catalytic amount of Et_3N (18 µL, 0.1 mmol) was added dropwise. The reaction mixture was stirred at r.t. for 16 h and after the crude was concentrated *in vacuo*. The product was purified by means of chromatography on silica gel with MPLC Syncore® Büchi eluting 0-100 % gradient of EtAOc in petroleum ether, as a transparent oil (594 mg, 2.16 mmol, 84% yield).

MS (ESI): 276 $[M+H]^+$, 298 $[M+Na]^+$.

^{1}H NMR (400 MHz, $CDCl_3$, δ ppm, *J* Hz): δ 7.47 (d, *J* = 7.9 Hz, 2H), 7.30 (d, *J* = 7.7 Hz, 2H), 4.66 (s, 2H), 4.15 (s, 2H), 3.99 (dd, *J* = 9.2, 4.1 Hz, 1H), 3.14 (dd, *J* = 18.8, 9.2 Hz, 1H), 2.67 (dd, *J* = 18.8, 4.2 Hz, 1H), 2.13 (s, 1H).

4-((2,5-dioxo-1-(prop-2-yn-1-yl)pyrrolidin-3-yl)thio)benzyl ((2*S*,3*S*,4*S*,6*R*)-3-hydroxy-2-methyl-6-(((1*S*,3*S*)-3,5,12-trihydroxy-3-(2-hydroxyacetyl)-10-methoxy-6,11-dioxo-1,2,3,4,6,11-hexahydrotetracen-1-yl)oxy)tetrahydro-2*H*-pyran-4-yl)carbamate (11)

9 (0.4 mmol) was solubilized in dry CH_2Cl_2 (5 mL) at r.t under N_2. At 0 °C *p*-nitrophenyl chloroformate (89 mg, 0.44 mmol) and DMAP (98 mg, 0.8 mmol) were added, and the reaction was stirred for 30 minutes at 0 °C. The activated compound was checked by TLC and was cannulated into a solution Doxorubicin (0.6 mmol) and DIPEA (209 µL, 1.2 mmol) at 0 °C. The reaction was stirred at r.t. for 16 h. The solvent was evaporated under reduced pressure, and the residue was dissolved in EtOAc (10 mL) and washed with H_2O (5 mL) and $NaCl_{SS}$ (5 mL). The organic phase was dried over dry Na_2SO_4, filtered, and evaporated under vacuum. The product was purified by flash chromatography (gradient $MeOH/CH_2Cl_2$ 0-5%) furnishing a red-orange solid (155 mg, 0.18 mmol) in 36% yield.

MS (ESI): 868 $[M+Na]^+$.

^{1}H NMR: (400 MHz, $CDCl_3$) δ 8.01 (d, J = 7.7 Hz, 1H), 7.76 (t, J = 8.1 Hz, 1H), 7.44 (d, J = 7.7 Hz, 2H), 7.37 (d, J = 8.5 Hz, 1H), 7.26 – 7.20 (m, 2H), 5.48 (d, J = 3.1 Hz, 1H), 5.27 (s, 1H), 5.13 (d, J = 8.6 Hz, 1H), 4.99 (d, J = 3.7 Hz, 2H), 4.74 (s, 2H), 4.52 (s, 1H), 4.14 (d, J = 2.5 Hz, 2H), 4.06 (s, 3H), 3.99 (d, J = 5.4 Hz, 1H), 3.84 (s, 1H), 3.65 (s, 1H), 3.47 (s, 2H), 3.26 (d, J = 19.4 Hz, 1H), 3.17 –

3.10 (m, 1H), 3.01 (d, J = 18.8 Hz, 2H), 2.68 – 2.63 (m, 1H), 2.32 (d, J = 15.0 Hz, 1H), 2.17 – 2.15 (m, 2H), 2.08 – 1.66 (m, 4H), 1.27 (d, J = 6.6 Hz, 3H).

6-(5-((3-((4-(((((2*S*,3*S*,4*S*,6*R*)-3-hydroxy-2-methyl-6-(((1*S*,3*S*)-3,5,12-trihydroxy-3-(2-hydroxyacetyl)-10-methoxy-6,11-dioxo-1,2,3,4,6,11-hexahydrotetracen-1-yl)oxy)tetrahydro-2*H*-pyran-4-yl)carbamoyl)oxy)methyl)phenyl)thio)-2,5-dioxopyrrolidin-1-yl)methyl)-1*H*-1,2,3-triazol-1-yl)hexanoic acid (12)

11 (30 mg, 0.03 mmol) and the 6-azidohexanoic acid (4 mg, 0.03 mmol) were dissolved in DMF dry (2 mL) under Ar. The solution was degassed with three cycles of argon/vacuum. To this solution, a freshly prepared aqueous mixture (1.5 mL) of $Cu(OAc)_2$ (1.6 mg, 0.01 mmol) and Na ascorbate (18 mg, 0.02 mmol), previously degassed by argon/vacuum cycles, was added dropwise. The reaction mixture was degassed and left to stir under Ar. at r.t. for 16 h. The solvent was evaporated and the crude was purified by silica gel flash chromatography eluting 0-10 % gradient of MeOH in CH_2Cl_2 provide the desired compound. A red solid (16 mg, 0.02 mmol) was obtained in 58% yield and directly used for the bioconjugation.

MS (ESI): 1002 $[M+H]^+$.

ADCs B242 and B238: bioconjugation reaction.

The proper carboxylic acid (**7** -for **ADC 242**- or **12** -for **ADC 238**) (0.01 mmol) was dissolved in dry DMF (0.5 mL) under N_2. DCC (3 mg, 0.014 mmol) and *N*-hydroxysuccinimide (2 mg, 0.014 mmol) were added to and the mixture was stirred at rt for 16 h under N_2. The solution was filtered and the solvent removed under vacuum obtaining a white solid that was dissolved in DMSO in order to obtain a 10 mM solution. Contemporary, a solution of Trastuzumab was buffer exchanged using a 10 kDa cutoff dialysis membrane to obtain the mAb in PBS pH 7.4 and to remove interfering preservative (glycine). The concentration of Trastuzumab after dialysis was determined measuring the OD280 and the observed absorbance was divided by 1.35. A 20-fold molar excess of the v NHS ester 10 mM solution was added to the dialyzed antibody solution. The reaction was incubated at room temperature with gentle continuous mixing and after 1 hour quenched with a 20 mM glycine aqueous solution. The final product was dialyzed in PBS at 4 °C using a 10 kDa cutoff membrane to remove the excess of unreacted payload.

A fraction of the final product was dialyzed against pure water for DAR determination via MALDI mass spectrometry (MALDI -TOF/TOF Bruker UltrafleXtreme) applying the following formula:

$$\text{DAR} = \frac{[\text{MW ADC B242 or B238}] - [\text{MW Trastuzumab}]}{[\text{MW 7 or 12} - H_2O]}$$

The obtained DAR for ADC B242 and ADC B238 were reported in the following table.

Sample	MW linker-payload	MALDI monocharged peak M^+	MALDI bicharged peak M^{2+}	Mean DAR
Trastuzumab		148548 Da	74305 Da	
ADC **B242**	784	150331 Da	75274 Da	2.3
ADC **B238**	1002	149867 Da	74937 Da	1.3

Linker-payload assignment in PBS of ADC B242.

The one-dimensional ^{1}H NOESY spectrum of the linker-payload system is reported in **Figure S2**. The assignment performed on the basis of ^{1}H-^{1}H TOCSY experiments, peak multiplicities and chemical shift predictions is summarized in the same figure, with a letter coding (a-z).

The one-dimensional spectrum and TOCSY spectra show evidence of multiple species (apices ‘, ‘’ in letters in **Figures S2-S7**). In the aromatic region of the spectrum, two singlet peaks at 8.31 and 7.85 ppm (signals a and b) in Figure S2, are complemented by a minor set of signals at 8.27 and 7.78 ppm (signals a’ and b’), in a ca 2:1 ratio. This is consistent with the HPLC of **Figure S8** and supports the presence of a mixture of diastereomers. The two singlet peaks are attributed to >CH- groups in between the nitrogen atoms. The same behaviour is observed in the TOCSY spectrum, where, in the aromatic region of the spectrum, two 4x4 patterns are observed for signals c-f and c’-f’ (see **Figure S3**). Additionally, the two >CH- groups of the pyrrole are observed in the region 6.8-6.60 ppm: indeed, we identify for the major component two signals almost overlapping at 6.80 ppm and 6.81 ppm, while another configuration is observed at 6.77 and 6.67 ppm, with a strong TOCSY connectivity, as shown in Figure S3.

The >CH-CH_3 close to the amino-phosphate group gives a single TOCSY peak that can be identified due to the peculiar downfield shift of the methyl signal, predicted to be in the range 1.4-1.10 ppm and observed as a doublet at 1.15 ppm. The doublet, indicate as signal u, is coupled with the multiplet at 3.45 ppm indicate as signal q, with intensity consistent with a single proton (see **Figure S4**). Another

TOCSY crosspeak is observed at 1.32 ppm (u') and 3.81 ppm (q'), which arises from another configuration of the >CH-CH_3 moiety.

In the aliphatic region of the spectrum, a 5x5 TOCSY pattern identifies the esanoic acid component of the linker-payload system (see **Figure S5**). The five signals at 4.53 ppm, 2.22 ppm, 2.00 ppm, 1.64 ppm and 1.37 ppm, labelled j, s, v, t and r in Figure S2, are individually assigned according to their multiplet pattern.

The analysis of signal at 0.75 ppm, attributed to the two equivalent methyl (-CH_3) groups labelled as z, reveals the presence of three different forms (z, z' and z''). Indeed, the observed multiplet can be simulated with three triplet signals, with very similar intensity, centered at 0.746 ppm, 0.750 ppm and 0.767 ppm (data not shown). Three different shifts are also observed for the >CH- next to the ester group and labelled as o, o' and o''. For these signals, three well separated shift values are observed at 3.81 ppm, 3.88 ppm and 3.94 ppm,

We identify a spin system via TOCSY spectra involving the methyl signal at 0.75 ppm and multiplet signals at 1.19 ppm (x), 1.34 ppm (w), 3.81 ppm (o), 3.88 ppm (o') and 3.95 ppm (o'') (see **Figure S6**). The analysis shows that the three signals o, o' and o'' are not aligned when considering the cross peaks with the methyl signals and also with the methylene (-CH_2-) signal x, as seen by the TOCSY. The resulting picture is a 4x4 pattern, as expected for the alkyl group attached to the carboxylic acid group.

NMR characterization of ADC B238.

A sample of the ADC B238 in PBS with a concentration of ~7 mg/mL was used to acquired methyl-edited ^{1}H -^{13}C-ALSOFAST-HMQC experiment using the same experimental parameters reported in the main text for free Trastuzumab and ADC B242. The low concentration was dictated by the limited solubility of this ADC. **Figure S8** shows that even at this low concentration, which is close to the detection limits for some signals, it is possible to establish a strong correlation (r=0.92) between the binned NMR spectra of free Trastuzumab and the ADC. Additionally, the same general behaviours (CCSD, broadening beyond detection and decrease in signal intensity) are observed. However, at this low concentration some signals could become undetectable due to the lower signal-to-noise ratio; at the same time the lower quality of the 2D map introduce larger errors in the measurement of chemical shifts. For all these reasons we consider this sample concentration as the lower limit for accurate measurement of ADC's HOS.

SUPPLEMENTARY FIGURES

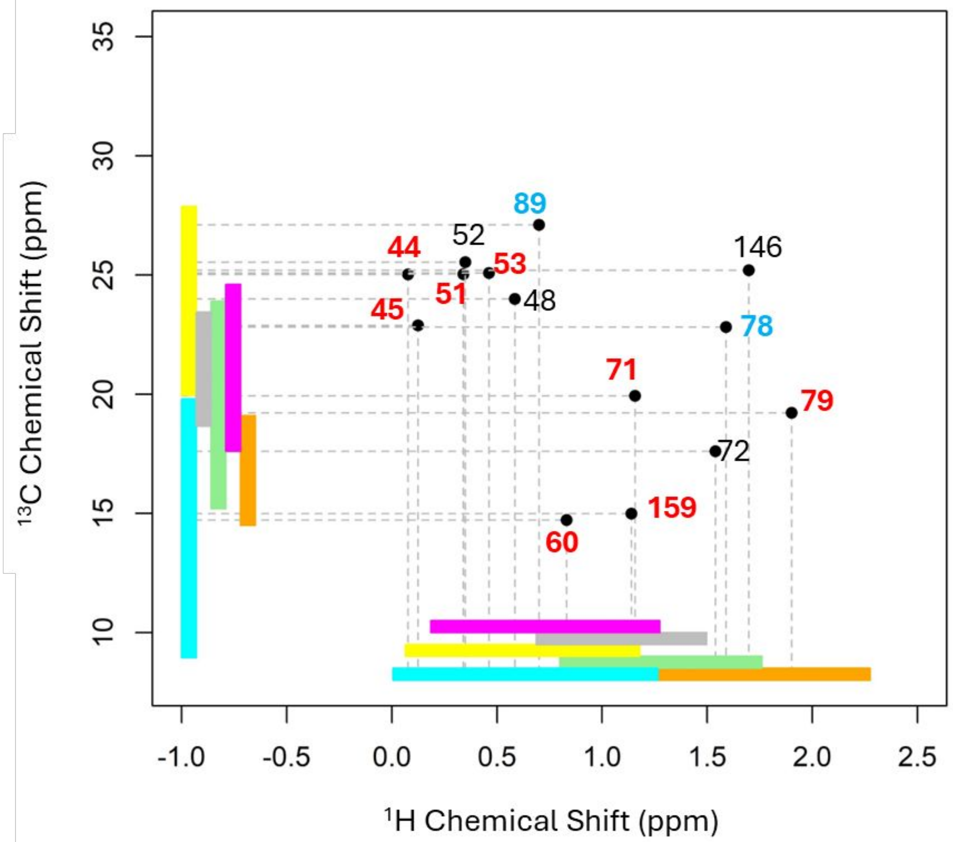

Figure S1. Putative assignment of the most affected signals listed in Figure 4, using the chemical shift intervals occurring in BMRB for the specific residues with frequencies greater than or equal to 5% of the modal class value.

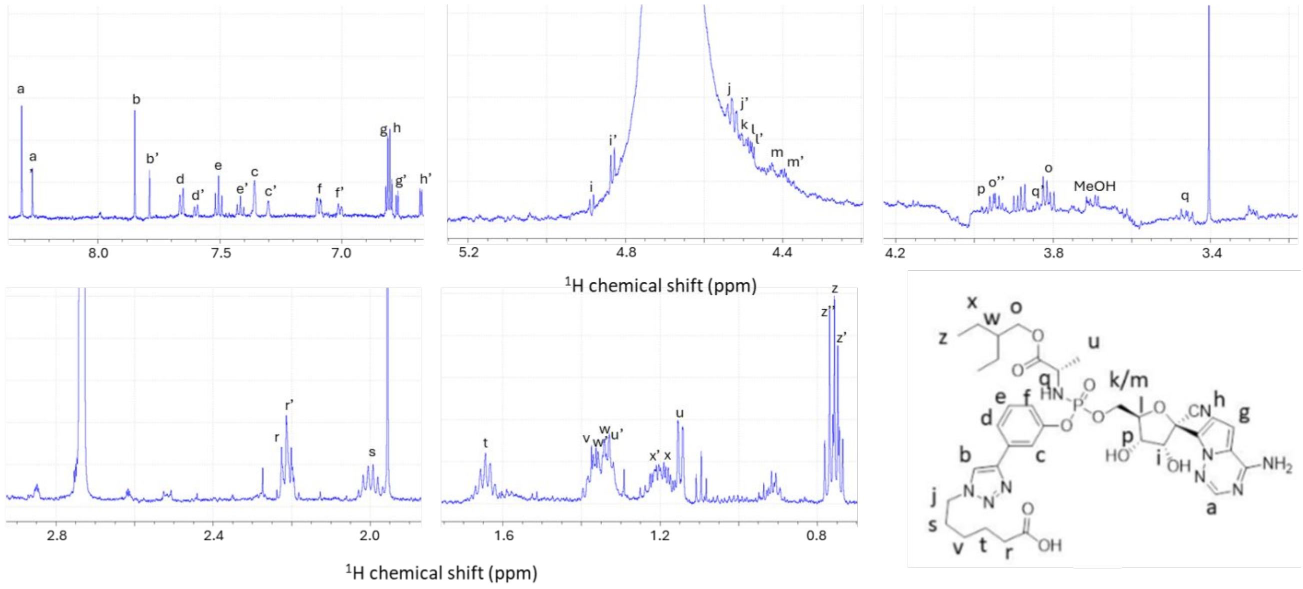

Figure S2. 1D spectrum of the linker-payload system, recorded in PBS at 600 MHz, with a noesy1d sequence. Selected portions of the spectra are expanded to highlight all signals in the spectrum. The proposed assignment is reported. Apices and double apices indicate different forms (e.g.: z, z', z'').

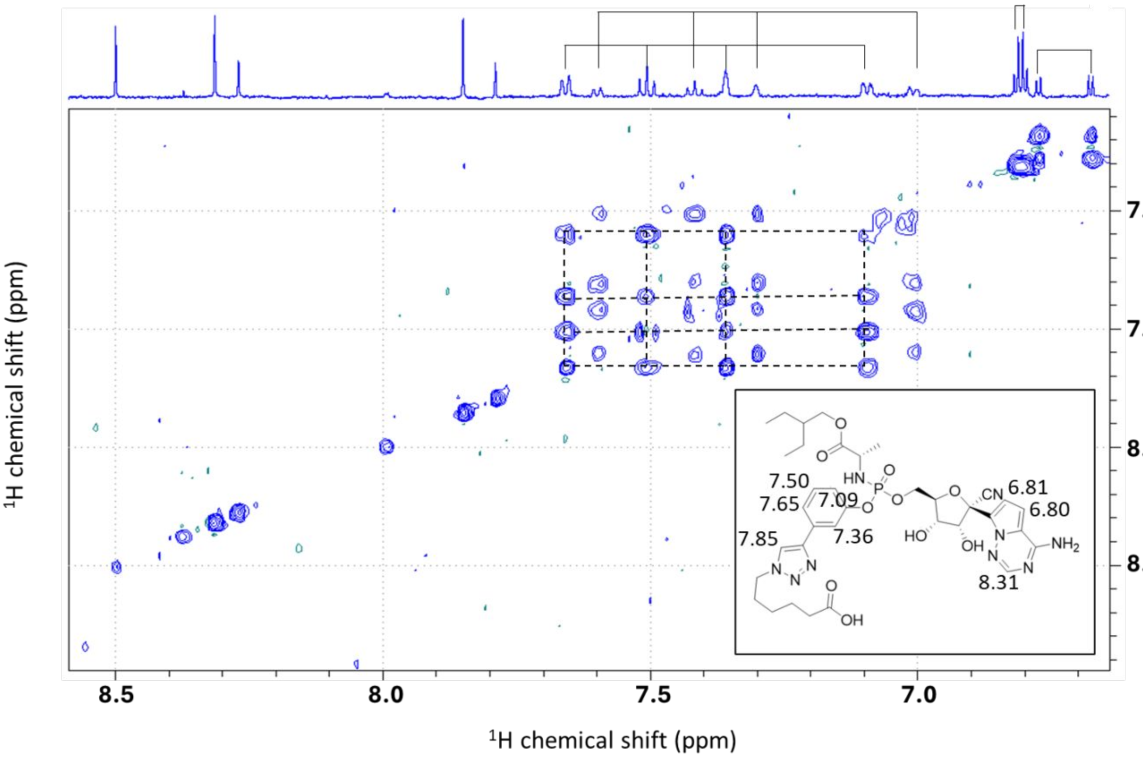

Figure S3. Aromatic part of the TOCSY spectrum (120 ms mixing time). Patterns are identified by dashed lines. The chemical shifts reported in the figure refers to the predominant configuration.

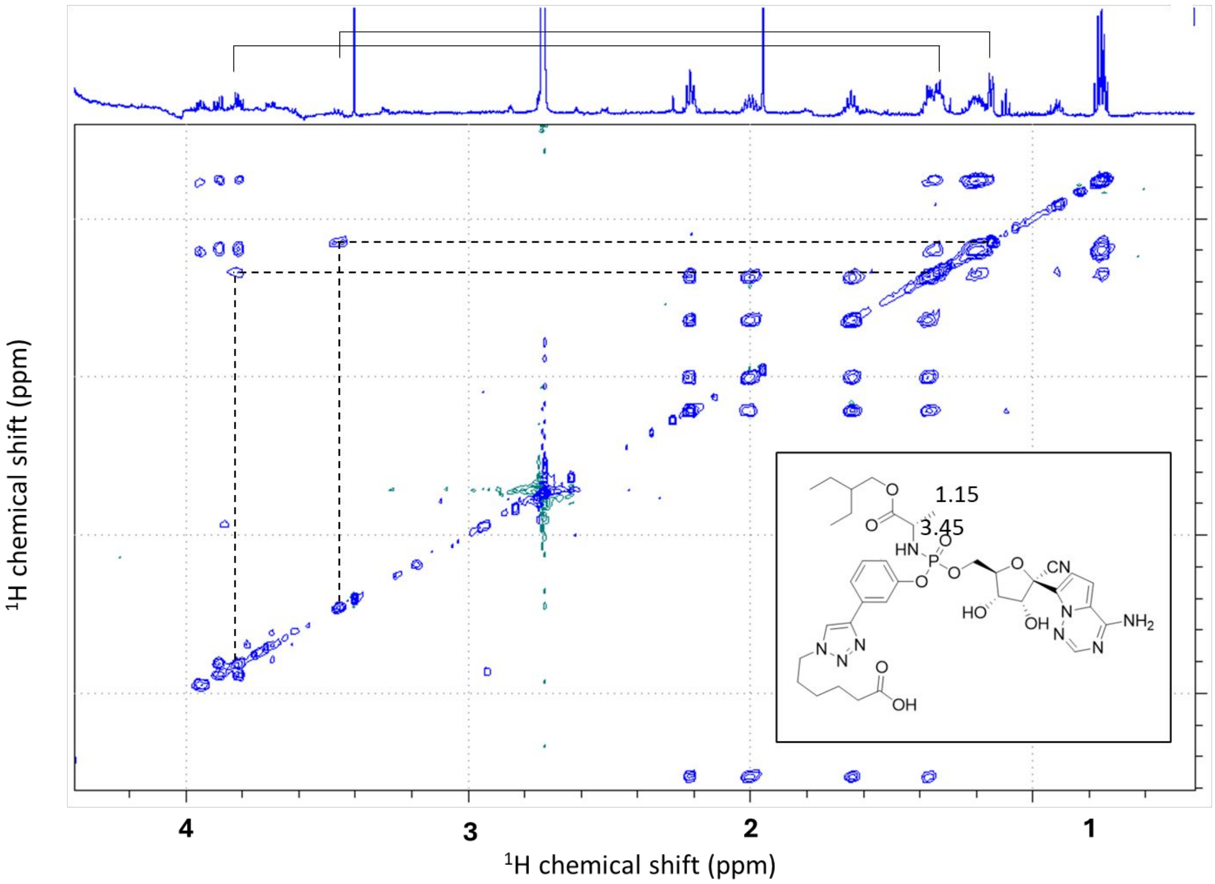

Figure S4. Sharp doublets at about 1.15 and 1.33 ppm. Their TOCSY connectivities are shown at 3.45 ppm and 3.84 ppm, respectively. In the inset the chemical shifts of the main form are reported.

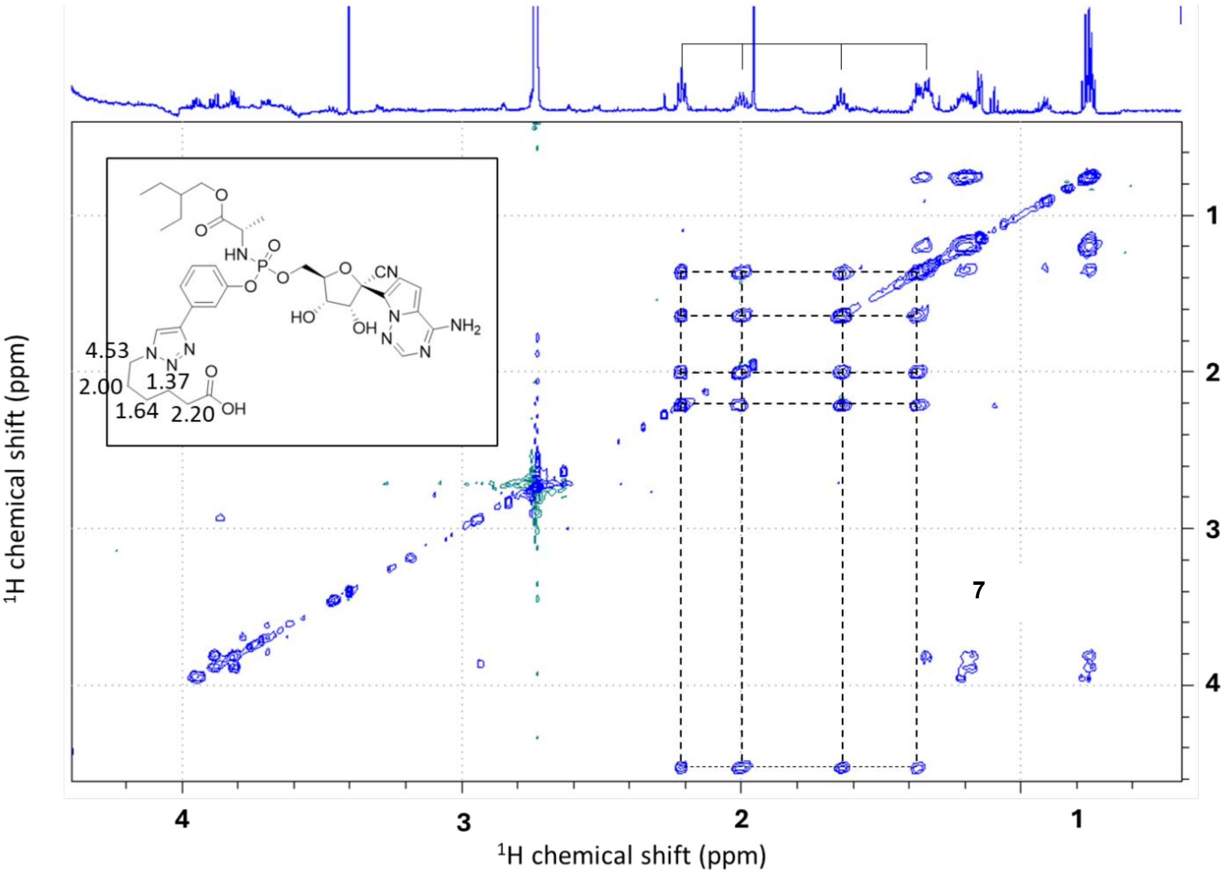

Figure S5. The TOCSY pattern of the flexible esanoic acid region.

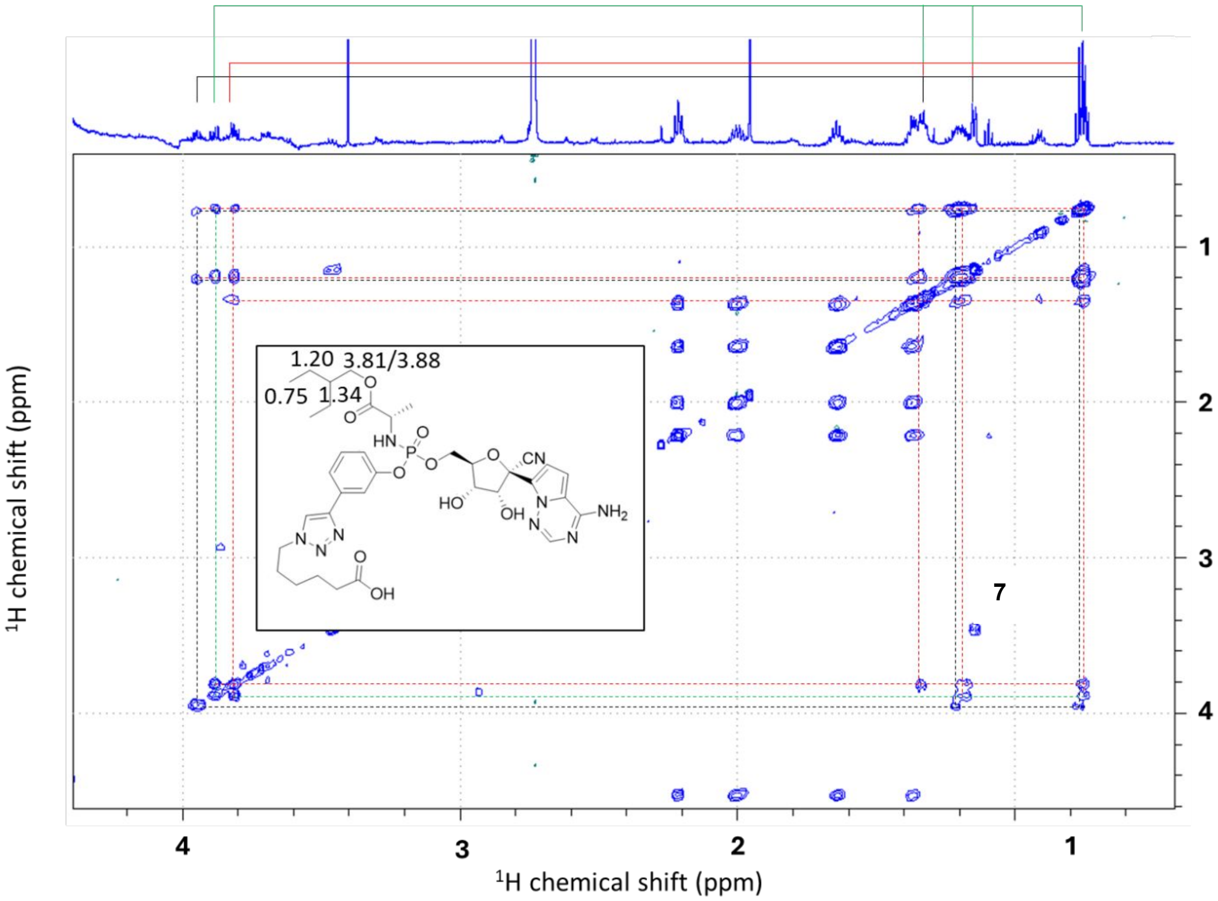

Figure S6. Three different forms are observed in the TOCSY patterns of the alkyl part of the molecule.

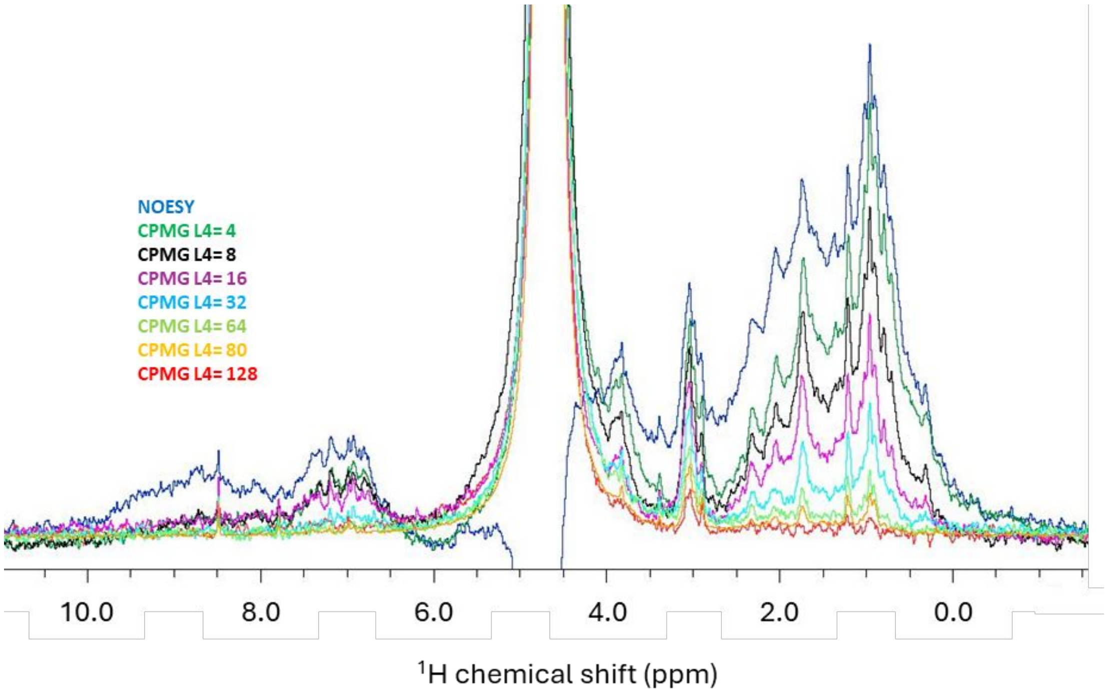

Figure S7. ^{1}H NMR spectra of free trastuzumab (14.4 mg/mL) acquired using a 600MHz spectrometer at 310K: comparison between 1D NOESY and 1D CPMG with different spin-lock times.

A

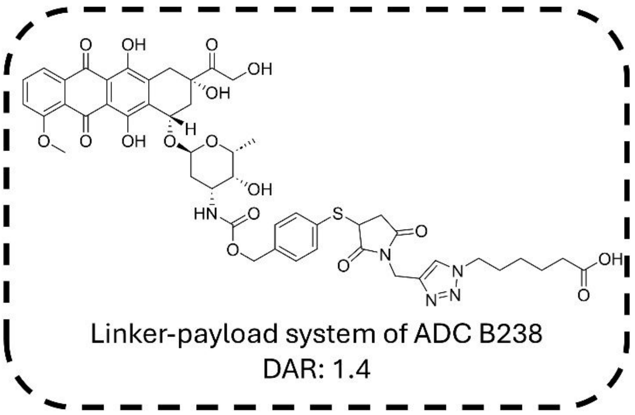

B

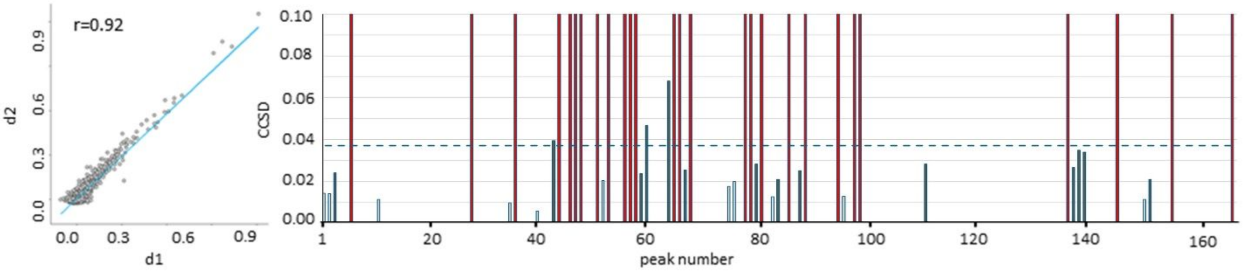

C

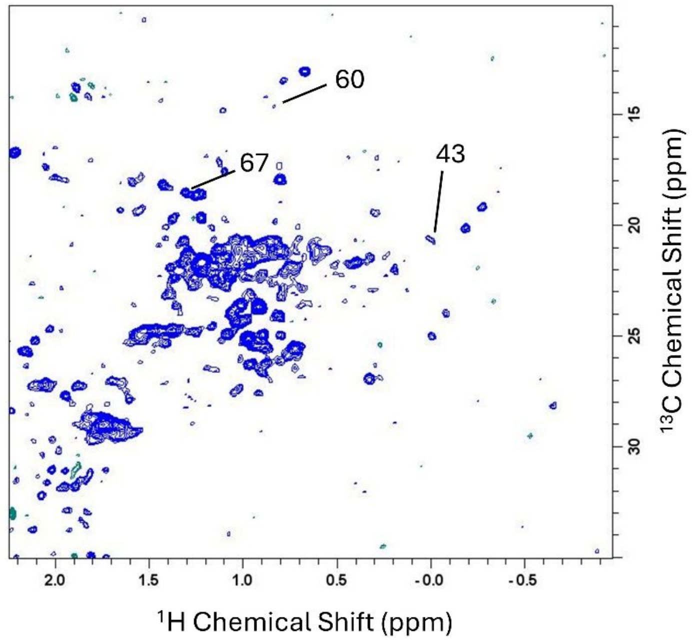

D

Peak number	Match with BMRB 52228	Peak number	Match with BMRB 52228
**Shift**			
43	V421	67	na
60	I53		
**Broad beyond detection**			
6	na	68	na
28	na	78	na
36	L22	79	na
44	L87	81	na
46	V247	86	na
47	na	89	L88
48	L133	95	na
51	L133	98	na
53	na	99	na
56	V66	137	na
57	I319	146	na
58	I273	156	na
65	na	165	L184
66	na		

Figure S8. ADC B238. A) Structure of the linker-payload system. B-D) Comparison between free Trastuzumab and ADC B242. B) Correlation analysis using binned NMR spectra (left panel) and combined chemical shift difference (CCSD) analysis, reported as bar plot (right panel) with CCSD's threshold (dashed line) taken as mean + standard deviation (0.037 ppm); CCSD values above the threshold indicate significant changes (dark grey bars) whereas values below the threshold are identified by light grey bars. The red bars represent the methyl peaks that disappeared in the spectrum

of ADC B238. C) Methyl-edited ^{1}H–^{13}C ALSOFAST-HMQC spectrum of ADC B238 acquired using a 950 MHz spectrometer at 310K. The most affected peaks in terms of chemical shift changes are labelled with the respective peak numbers. D) Table listing the most affected peaks with the match with BMRB 52228 assignment (2nd column).

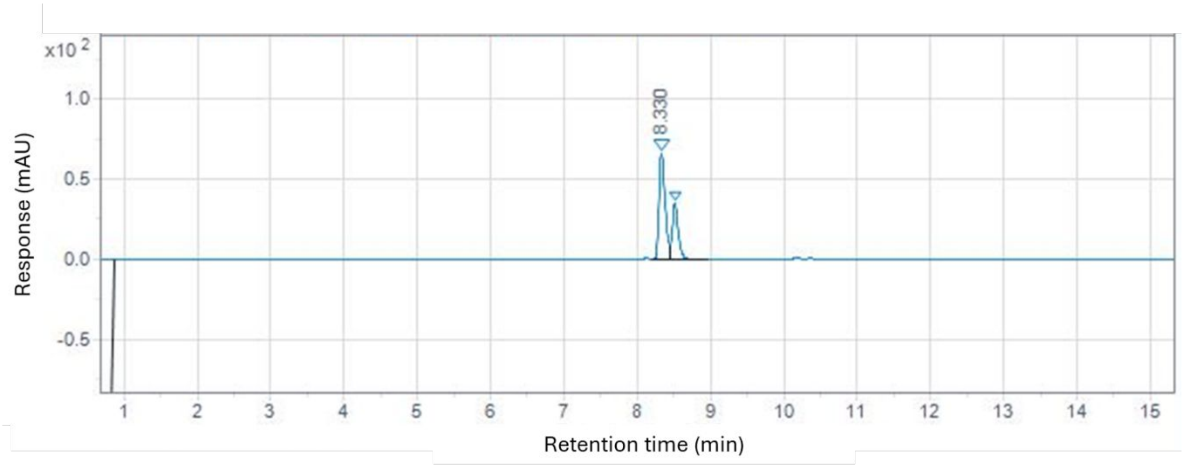

Figure S9. HPLC of diastereoisomers mixture of compound **7.**

SUPPLEMENTARY TABLES

Table S1. *Assignment of methyl groups of Isoleucine, Leucine and Valine based on the sequence-specific assignment of the single chain Fab fragment of Trastuzumab reported in BMRB 52228 entry.*

Amino acid	Assignment	Match	Amino acid	Assignment	Match
V4	QG1/CG1	X	L266	QD2/CD2	
V4	QG2/CG2		L266	QD1/CD1	
L6	QD1/CD1	X	V267	QG2/CG2	X
L6	QD2/CD2		V267	QG1/CG1	X
V7	QG2/CG2		I273	QD1/CD1	X
V7	QG1/CG1		L282	QD1/CD1	
L13	QD2/CD2		L282	QD2/CD2	X
L13	QD1/CD1	X	V286	QG2/CG2	
V14	QG1/CG1		V286	QG1/CG1	
V14	QG2/CG2		V290	QG1/CG1	
L22	QD2/CD2		V290	QG2/CG2	
L22	QD1/CD1	X	I292	QD1/CD1	X
I31	QD1/CD1	X	V300	QG2/CG2	X
I36	QD1/CD1	X	V300	QG1/CG1	
L47	QD2/CD2		V304	QG1/CG1	
L47	QD1/CD1	X	V304	QG2/CG2	
V50	QG2/CG2		L317	QD1/CD1	
V50	QG1/CG1	X	L317	QD2/CD2	
I53	QD1/CD1	X	L318	QD1/CD1	X
V66	QG1/CG1	X	L318	QD2/CD2	
V66	QG2/CG2	X	I319	QD1/CD1	X
I72	QD1/CD1		L325	QD1/CD1	
L83	QD2/CD2	X	L325	QD2/CD2	
L83	QD1/CD1	X	V329	QG1/CG1	X
L88	QD1/CD1	X	V329	QG2/CG2	X
L88	QD2/CD2		L344	QD2/CD2	
V95	QG1/CG1		L344	QD1/CD1	X
V95	QG2/CG2	X	I346	QD1/CD1	
L117	QD2/CD2		L349	QD1/CD1	X
L117	QD1/CD1		L349	QD2/CD2	
V118	QG1/CG1	X	V375	QG1/CG1	
V118	QG2/CG2		V375	QG2/CG2	
V120	QG1/CG1	X	I377	QD1/CD1	
V120	QG2/CG2		V381	QG1/CG1	
V130	QG1/CG1		V381	QG2/CG2	X
V130	QG2/CG2		V386	QG1/CG1	
L133	QD1/CD1	X	V386	QG2/CG2	
L133	QD2/CD2	X	I388	QD1/CD1	X
L147	QD1/CD1		L396	QD1/CD1	X
L147	QD2/CD2		L396	QD2/CD2	X

L150	QD1/CD1	X	V403	QG2/CG2	
L150	QD2/CD2		V403	QG1/CG1	
V151	QG1/CG1		V404	QG2/CG2	
V151	QG2/CG2		V404	QG1/CG1	
V159	QG1/CG1	X	L406	QD1/CD1	
V159	QG2/CG2		L406	QD2/CD2	
V161	QG2/CG2		L407	QD1/CD1	
V161	QG1/CG1		L407	QD2/CD2	
L168	QD1/CD1	X	V417	QG1/CG1	
L168	QD2/CD2		V417	QG2/CG2	X
V172	QG1/CG1		V421	QG2/CG2	
V172	QG2/CG2		V421	QG1/CG1	X
V178	QG1/CG1		L425	QD1/CD1	
V178	QG2/CG2		L425	QD2/CD2	X
L179	QD1/CD1	X	V434	QG2/CG2	
L179	QD2/CD2	X	V434	QG1/CG1	X
L184	QD1/CD1	X	L446	QD2/CD2	X
L184	QD2/CD2	X	L446	QD1/CD1	X
L187	QD1/CD1	X	L450	QD1/CD1	X
L187	QD2/CD2	X	L450	QD2/CD2	X
V190	QG2/CG2	X	L452	QD1/CD1	X
V190	QG1/CG1		L452	QD2/CD2	X
V191	QG1/CG1	X	V462	QG1/CG1	
V191	QG2/CG2	X	V462	QG2/CG2	X
I204	QD1/CD1	X	V467	QG2/CG2	X
V207	QG1/CG1		V467	QG1/CG1	
V207	QG2/CG2		L472	QD1/CD1	X
V216	QG2/CG2	X	L472	QD2/CD2	
V216	QG1/CG1		V476	QG1/CG1	X
V220	QG1/CG1	X	V476	QG2/CG2	X
V220	QG2/CG2				

Table S2. *Representative ^{13}C and ^{1}H chemical shift regions for the methyl groups of each amino acid (mean ± standard deviation), taken from BMRB statistics.*

	**^{13}C**		**^{1}H**	
Amino acid	Mean	Standard deviation	Mean	Standard deviation
	CB		QB	
**ALANINE**	19.029	2.876	1.353	0.275
	CD1		QD1	
**ISOLEUCINE**	13.487	3.294	0.674	0.325
	CG2		QG2	
	17.598	3.128	0.770	0.301
	CD1		QD1	
**LEUCINE**	24.651	2.004	0.747	0.326
	CD2		QD2	
	24.131	2.078	0.729	0.382
	CE		QE	
**METHIONINE**	17.238	3.992	1.787	1.469
	CG2		QG2	
**THREONINE**	21.591	1.843	1.139	0.272
	CG1		QG1	
**VALINE**	21.528	2.330	0.819	0.330
	CG2		QG2	
	21.344	2.426	0.802	0.417
